# Usability analysis of a web laboratory for cognitive stimulation with acquired brain injury users

**DOI:** 10.1002/alz.091586

**Published:** 2025-01-09

**Authors:** Maria Celeste López Moreno, Karen D Urriategui, Axel Fernández‐Zaionz, Elsa Araceli Revollo Sarmiento, Leticia Vivas

**Affiliations:** ^1^ IPSIBAT (UNMDP/CONICET), Mar del Plata, Buenos Aires Argentina; ^2^ Universidad Nacional de Córdoba, Córdoba, Córdoba Argentina; ^3^ IPSIBAT (CONICET‐National University of Mar del Plata), Mar del Plata Argentina; ^4^ Universidad Nacional de Mar del Plata, Mar del Plata, Buenos Aires Argentina; ^5^ IPSIBAT (UNMDP‐CONICET), Mar del Plata, Buenos Aires Argentina; ^6^ IPSIBAT (CONICET/National University of Mar del Plata), Mar del Plata, Buenos Aires Argentina

## Abstract

**Background:**

In 2020, we developed LABPSI, a cognitive stimulation web lab. Usability analysis in MCI and healthy participants have already been studied, and currently, we performed it with acquired brain injuries (ABI) participants, as they can rehabilitate their cognitive symptoms and prevent the progression to dementia. Usability can be considered the ease of use of a certain product for a specific aim by a particular population. The study’s primary objective is to assess LABPSI’s long‐term usability in ABI participants.

**Method:**

Our sample comprised 12 ABI participants with focal and diffuse lesions (33.3% stroke, 41.7% TBI, 25% others) in the subacute phase. All participants were in treatment during data collection. With a mean age of 51.08 (SD = 19.96, range: 24‐77 years), we recorded their educational level (average: 9.91 years) and technological expertise (mean: 80.41) regarding the maximum questionnaire score of 136 (Range = 49‐109; SD = 19.29), it reflects a moderate to high level of technology familiarity. LABPSI underwent a long‐term assessment using a pre‐experimental design with a single group, including pre‐test, guided training, and post‐test phases. Objective measures evaluated effectiveness and efficiency of a specific task on the LABPSI, which was divided into: initial task phase (steps from the beginning of LABPSI to the start exercises) and exercise resolution phase. The effectiveness was operationalized through steps achieved and the number of errors, while the efficiency considers the time spent to achieve the task and steps achieved in 3 minutes.

**Result:**

A Wilcoxon Test indicated significant results in 5 out of 7 measures. In the initial task phase, time spent (p = .380) and errors (p = .463) were not significant, but steps achieved and steps achieved in 3 minutes were (p<.05). For the exercise resolution phase, all objective metrics (steps achieved, errors, and time spent) showed significant values (p<.05) indicating better performance in the post‐test.

**Conclusion:**

Our findings suggest that LABPSI exhibits promise as a rehabilitative tool for individuals with ABI, featuring a user‐friendly interface tailored to their needs. Exploring the long‐term usability of LABPSI enhances our understanding and contributes to knowledge on cognitive rehabilitation strategies, aiding in collaborative efforts to prevent dementia.

